# CSA-Net: Channel and Spatial Attention-Based Network for Mammogram and Ultrasound Image Classification

**DOI:** 10.3390/jimaging10100256

**Published:** 2024-10-16

**Authors:** Osama Bin Naeem, Yasir Saleem

**Affiliations:** 1 Department of Electrical Engineering, University of Engineering and Technology, Lahore-Narowal Campus, Narowal 51600, Pakistan; 2 Department of Computer Engineering, University of Engineering and Technology, Lahore 39161, Pakistan; yasir@uet.edu.pk

**Keywords:** attention mechanism, breast cancer, binary classification, classification, deep learning, mammogram, ultrasound, mass, multi-classification

## Abstract

Breast cancer persists as a critical global health concern, emphasizing the advancement of reliable diagnostic strategies to improve patient survival rates. To address this challenge, a computer-aided diagnostic methodology for breast cancer classification is proposed. An architecture that incorporates a pre-trained EfficientNet-B0 model along with channel and spatial attention mechanisms is employed. The efficiency of leveraging attention mechanisms for breast cancer classification is investigated here. The proposed model demonstrates commendable performance in classification tasks, particularly showing significant improvements upon integrating attention mechanisms. Furthermore, this model demonstrates versatility across various imaging modalities, as demonstrated by its robust performance in classifying breast lesions, not only in mammograms but also in ultrasound images during cross-modality evaluation. It has achieved accuracy of 99.9% for binary classification using the mammogram dataset and 92.3% accuracy on the cross-modality multi-class dataset. The experimental results emphasize the superiority of our proposed method over the current state-of-the-art approaches for breast cancer classification.

## 1. Introduction

There are 37.2 trillion cells in the human body [1], which undergo a continuous cycle of division, proliferation, and eventual death. This process undergoes strict regulation and continuous operation throughout life. On average, the human body replaces its cells every seven to ten years, although the lifespan of specific organs may vary [2]. When ageing, if cells fail to undergo programmed cell death and instead continue to divide and proliferate uncontrollably, they can form tumors. However, there is an exception in the case of leukemia, which does not produce tumors. This condition poses a serious health risk and could lead to life-threatening consequences [3].

**Figure 1:**
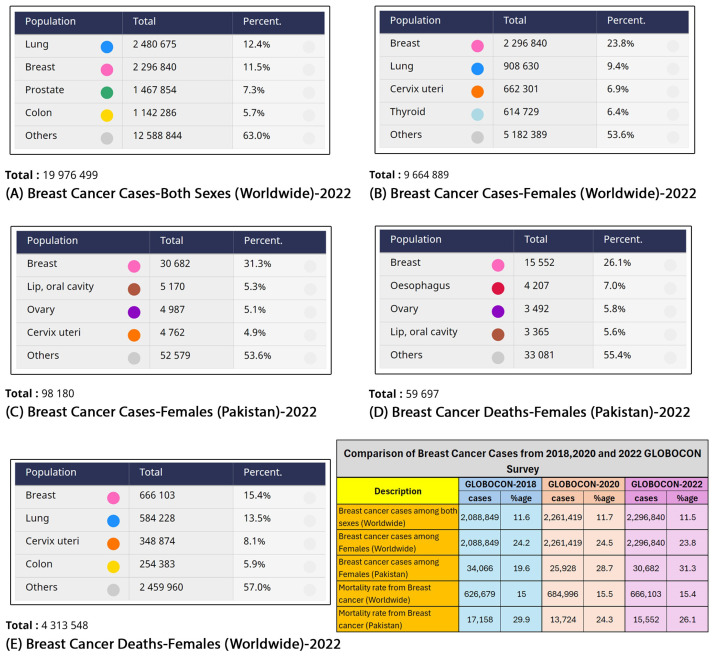
Cancer cases according to GLOBOCON in 2018, 2020, and 2022—WHO [4,5,7,8].

Breast cancer ranks among the most common cancers worldwide in women, although its incidence varies from region to region. According to the World Health Organization’s (WHO) 2022 report and research conducted by the International Agency for Research on Cancer, cancer stands as the leading cause of abnormality and mortality globally, affecting both genders; see Figure 1A. In Figure 1B, the distribution of female cancer cases globally in 2022 is depicted in terms of the percentile distribution. Women represent the majority of the reported instances, accounting for 23.8% (2,296,840 cases) of the total. Additionally, Figure 1C documents these cancer cases in Pakistan, comprising 31.3% (30,682 cases) of all cancer cases [4,5].

Computer-aided detection (CAD) systems in mammography have historically faced performance challenges due to their reliance on radiometric features, leading to unnecessary treatments and costs. Deep learning (DL) promises to address these limitations, although it requires substantial training data. Medical imaging data, adhering to big data principles, presents both opportunities and hurdles. DL has the potential to improve performance and reduce costs through enhanced initial screenings. Ultimately, DL could improve early breast cancer diagnosis and healthcare efficiency [6].

Despite the prevalence of breast cancer, traditional detection methods fall short in terms of performance compared to emerging technologies like deep learning. The application of deep learning in medical imaging, particularly for breast cancer detection, is hindered by the lack of large-scale training data. Although deep learning shows promise in pattern recognition through neural network layers, its effectiveness in this domain is constrained by data availability. Therefore, there is a significant opportunity to enhance screening accuracy through further research and development of automated breast cancer detection systems [9]. Our study aims to contribute to these efforts by utilizing deep learning models for the classification of breast abnormalities, thereby supporting ongoing endeavours to improve cancer screening efficiency and accuracy.

The proposed research architecture assimilates the pre-trained EfficientNet-B0 model, renowned for its ability to extract intricate features from diverse datasets. By integrating channel and spatial attention mechanisms, the model enhances its discriminative capabilities, focusing on salient features that are crucial for accurate breast cancer classification.

Channel and spatial attention are used as a convolutional block attention module (CBAM), allowing the model to concentrate on relevant spatial regions and effectively address class imbalances. Overall, this comprehensive approach aims to elevate the performance in the complex task of breast cancer classification by strategically incorporating attention mechanisms and focal loss.

This paper is organized into sections as follows. The literature work is cited in Section 2. Then, Section 3 covers the methodology used in obtaining the desired results. Section 4 covers the results, where Section 4.1 covers the datasets used, the system configuration used for the experimentation, and the evaluation measures, and Section 5 covers the analysis conducted in response to the experimentation conducted. Finally, Section 6 displays the conclusions in response to the results generated.

## 2. Related Work

Deep neural networks are widely explored and applied in breast cancer diagnosis due to their robust feature extraction capabilities. Numerous methods utilizing convolutional neural networks (CNNs) for feature extraction have been introduced to enhance the efficiency of breast cancer diagnosis [10,11,12].

Two main strategies have emerged to improve the efficiency of breast cancer detection with CNNs, capitalizing on their capability to learn complex features and label data in detail, particularly in mammogram images. One approach integrates information from both the complete mammogram image and labeled image patches. A deep fusion model is introduced to tackle anomaly detection in mammogram images by incorporating patch information alongside the broader image context. This method entails two key steps: initially, region of interest (ROI) images are trained, followed by investigating the channel’s interrelationships within the block [13].

In [14], the authors proposed a method to improve the classification accuracy, where the CNN was initially pre-trained using patch data, followed by fine-tuning on complete mammography images. While this approach proved to yield more detailed lesion characteristics, it required additional annotations for patch labels. In [15], the authors introduced another approach that integrated both cranio-caudal (CC) and medio-lateral oblique (MLO) views, along with their corresponding segment annotations of the same breast, as input to neural networks. This method aimed to enhance the classification of mammogram images. Additionally, they proposed an automated approach to classifying MLO views and unregistered CC views. This method improved the effectiveness of breast cancer diagnosis by integrating images from both views with their corresponding segmentation maps of breast lesions [16].

Ref. [17] compiled a substantial dataset of mammography images to train a deep CNN designed for breast cancer diagnosis. Each examination encompassed four distinct image views, and the deep CNN utilized these comprehensive examinations as input to predict the potential class. This approach is advantageous as it integrates a larger amount of input data, thereby enhancing the performance. Both strategies contributed to the improved effectiveness of breast cancer diagnosis by harnessing additional data, such as multi-view and patch label information. However, it is noteworthy that higher-grade datasets might necessitate more supplementary information. Additionally, the process of compiling and annotating a high-quality medical dataset requires significant effort, time, and specialized expertise.

### 2.1. CNN-Based Models

In recent studies, several researchers have proposed that enhancing the feature extraction process could improve breast cancer diagnosis. By focusing on the extraction of discriminating features, these techniques aim to enhance the output while reducing the need for manual labor. For example, researchers proposed a group-max pooling structure based on regions to efficiently extract discriminating features from the data [18]. The authors in [19] implemented a waterfall technique using generative and discriminative approaches to obtain discriminating features. In this method, abnormal tissue was treated as an outlier, while normal tissue was accurately modeled. Enabling the feature extraction component to collect discriminative features from images, several researchers redesigned the loss function from conventional softmax to an angular space and integrated loss restraints. For instance, ref. [20] pioneered the SphereFace technique, which utilizes a hyperparameter *m* to regulate the distribution of various classes using a novel loss function. The hyperparameter *m* defines the dimensions of the distribution space. Building upon this concept, refs. [21,22] developed ArcFace and CosFace, expanding on these principles.

Although initially developed for face recognition tasks, the direct application of these methods to mammogram image classification has led to a significant decrease in effectiveness. Yan Wang et al. extended the utilization of the angular softmax loss function from face recognition to mammography image classification in their research methods. This approach bore similarities to previous studies, such as the utilization of dropout-based Bayesian uncertainty measures by Leibig et al. to enhance the detection of diabetic retinopathy from fundus images [23,24]. To improve the classification performance, the authors integrated calibration and uncertainty within the framework of Bayesian deep learning models [25].

Analyzing mammograms often leads to unnecessary biopsies, resulting in wasted time and resources. To address this issue, the authors in [6] utilized neural networks, along with techniques like noise reduction, thus benefiting both the patient and the healthcare system.

In [26], the researchers endeavored to categorize breast mammography images into normal, benign, and malignant classes. Morphological techniques were applied to adjust the image characteristics, while preprocessing methods like median and mean filters were implemented to enhance the image quality and mitigate noise.

The methodology outlined in [27] employed adaptive median filtering, edge detection, Sobel filtering, and histogram equalization to enhance the image quality and remove unwanted elements like pectoral muscles and impulsive noise. Transfer learning was then applied to extract relevant features from mammograms. Succeeding this, reduced dimensionality and principal component analysis (PCA) were utilized to extract features, balancing computational efficiency with information preservation.

In another study, the author [9] employed median and Wiener filters as filtering techniques and opted for contrast adaptive histogram equalization (CLAHE) due to its effectiveness in enhancing breast images. The classification was performed using a CNN model, ResNet50, with transfer learning applied for feature extraction. Data augmentation techniques were utilized to expand the dataset.

### 2.2. Attention-Based CNN Models

Inspired by the human cognitive process of selectively filtering out irrelevant information to focus on important aspects, researchers have developed attention mechanisms in deep learning algorithms. These attention mechanisms mimic the human ability to prioritize and focus on essential components while processing large volumes of data. By dynamically allocating attention to specific parts of the input data, these algorithms effectively filter out noise and irrelevant details, allowing them to extract and emphasize the most relevant features. This approach enhances the efficiency and effectiveness of sorting through homogeneous datasets by directing the computational resources towards the most significant aspects of the data, similar to the way in which human attention operates in daily information exploration [28].

The CNN has proven its effectiveness in applications. Moreover, attention mechanisms, explored in image classification research, have been found to further improve the performance of CNNs [29].

In a recent study [30], the authors suggested integrating the ResNet network with a convolutional block attention module (CBAM) to tackle classification problems. The proposed model consisted of a classification module, attention mechanisms, and a backbone comprising ResNet structures. CBAM integration involved incorporating channel and spatial attention maps into ResNet blocks, enhancing the feature representation. The model also addressed the spatial characteristics of CT scan images, focusing particularly on the consolidations and ground glass opacities commonly observed in COVID-19 cases. This approach enabled the model to effectively capture both prominent and subtle features present in grayscale levels.

In [31], skin lesions were classified using the DenseNet-121 model, specifically chosen for its dense connections, facilitating the skip connection by the transmission of lesion features through the layers. DenseNet-121 employed various numbers of densely linked convolutional layers to extract multi-scale lesion features. To enhance the feature map information, class-wise attention was incrementally applied after specific dense blocks, chosen based on high-level semantic information. This attention mechanism enabled the learning of class-specific properties from regions before the final classification.

There has been a notable transition from manual feature extraction to the automated identification, selection, and classification of breast abnormalities. Deep learning algorithms have emerged as the preferred choice over traditional feature-based classifiers due to their numerous advantages, leading to widespread adoption in the biomedical field for breast cancer diagnosis [32,33]. In deep learning, the process of feature extraction and selection is seamlessly incorporated within the network architecture, eliminating the necessity for manual intervention. Consequently, deep learning approaches demonstrate enhanced capabilities in handling complex data and are regarded for their reliability. However, the intricate nature of deep neural networks often results in less interpretable outcomes, posing challenges in assessing the diagnostic significance of imaging data and the individual contribution of each feature to the classification performance.

The authors in [34] introduced a novel multi-scale convolutional neural network (CNN) to classify breast masses as benign or malignant. The architecture leverages two distinct CNN models: Res2Net for the extraction of global features from larger images and DenseNet for the capture of fine-grained details from smaller patches. Both networks were equipped with a convolutional block attention module (CBAM), which enhanced the model’s ability to focus on the most relevant features while disregarding less useful information. After feature extraction, the global and local features were merged in a fully connected layer to perform the final classification. This multi-scale approach enabled the model to integrate detailed and broader contextual information, significantly improving the accuracy and robustness of the classification task.

In [35], the INbreast dataset was used to classify breast masses as benign or malignant. They developed a custom preprocessing pipeline, the BDP method, which enhanced the image quality by applying noise reduction, contrast improvement through CLAHE, and data augmentation techniques. The classification was performed using an enhanced ResNet50 model, called ECA-Net50, which integrated an efficient channel attention (ECA) module to improve the feature extraction. To handle class imbalances and challenging samples, the focal loss was used during training, whereas transfer learning fine-tuned the pre-trained model on the mammogram dataset to optimize the performance further.

In [36], the author used thermal imaging from the DMR-IR dataset. The study involved preprocessing the dataset of grayscale thermal images by cropping, normalization, and resizing, followed by data augmentation to enhance the dataset’s diversity. A convolutional neural network (CNN) was utilized for feature extraction. Furthermore, a bidirectional long short-term memory (BLSTM) layer was introduced to leverage temporal information. At the same time, three types of attention mechanisms—self-attention, soft attention, and hard attention—were integrated to emphasize critical areas within the thermal images. The model’s efficiency was assessed, with the results indicating that the models incorporating attention mechanisms performed significantly better, indicating the importance of attention in enhancing breast cancer detection.

### 2.3. Breast Cancer Classification Challenges

Comparing the different techniques mentioned in the literature is challenging due to inconsistencies in reporting the outcomes. The accuracy, precision, sensitivity, specificity, F1-score, and area under the curve (AUC) are common evaluation metrics. It is essential for a paper to include all relevant performance evaluation measures to comprehensively describe an algorithm. Solely presenting the accuracy and AUC may be misleading, as high accuracy can be achieved by trading off low sensitivity and high specificity. However, in clinical settings, low sensitivity is undesirable as it correlates with a high rate of false negative detections.

The early diagnosis of breast cancer significantly reduces unnecessary stress by allowing for timely treatment interventions when the tumor is still small and localized. Research indicates that diagnosing breast cancer at an early stage improves the prognosis and reduces the overall medical costs by minimizing the need for extensive treatments and procedures later on [17,37,38,39,40,41,42]. Despite this, many patients are referred for biopsy even when the likelihood of a malignancy is low, leading to unnecessary surgical procedures that are not only painful but also carry negative psychological and financial burdens. Studies have shown that only a small percentage of these referred cases are malignant, thus highlighting the need for more accurate and efficient diagnostic methods to minimize unnecessary interventions and to improve patients’ care.

To improve the effectiveness of mammogram interpretation, it is imperative to enhance the diagnostic efficiency [38,41,42,43,44,45,46,47,48,49,50,51,52] while simultaneously reducing the occurrence of false alarms [38,40,47,49,50]. This approach aims to optimize the readability of mammograms by ensuring that legitimate abnormalities are correctly identified, while minimizing unnecessary concerns raised by false positive findings.

## 3. Methodology

This section presents an overview of the proposed methodology.

### 3.1. Proposed Architecture Overview

The CSA-NET architecture represents a sophisticated framework designed for precise breast cancer diagnosis by embedding advanced deep learning techniques and specialized attention mechanisms tailored to the analysis of mammograms and ultrasound images. The workflow begins with preprocessing steps applied to mammograms, which include standardization, resizing, and augmentation techniques. These steps are crucial in improving the dataset quality and class heterogeneity and essential for robust model training. These are important aspects, especially when facing the class imbalance challenge, which is often encountered in medical datasets and is addressed through strategies like upsampling.

**Figure 2:**
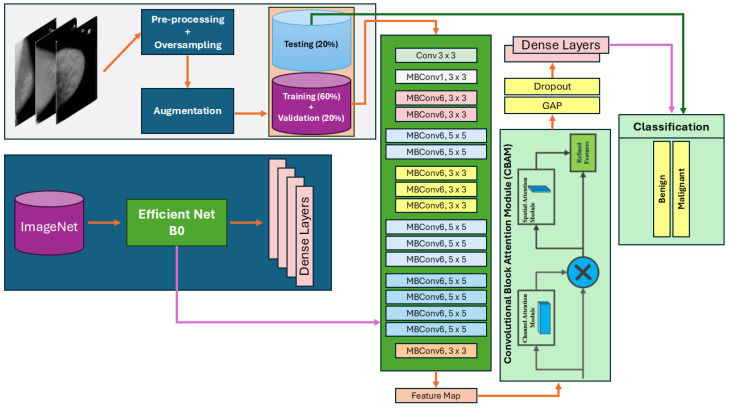
Proposed CSA-Net architecture.

Vital to CSA-NET’s architecture is the utilization of transfer learning with the EfficientNet-B0 model, which is selected based on a performance evaluation of different CNN models trained on the mammogram dataset, as evaluated in Table 1. Leveraging pre-trained models such as EfficientNet-B0 [53], initially pre-trained on ImageNet, provides a significant advantage by harnessing learned features from a large-scale dataset. By integrating attention mechanisms into the EfficientNet-B0 backbone, CSA-NET enhances its capacity to capture salient features from mammogram images effectively. Please refer to Figure 2.

The attention mechanisms in CSA-NET consist of two key components: channel attention and spatial attention. The channel attention mechanism optimizes the feature channel importance, allowing the model to prioritize the most discriminative aspects of the input mammogram images. This recalibration enhances the model’s ability to discern crucial features for accurate classification.

Following channel attention, the spatial attention mechanism further refines the feature representations by highlighting pertinent spatial regions within the mammogram images. This refinement process enhances the model’s sensitivity to diagnostically relevant areas while minimizing noise from less informative regions. Such hierarchical attention mechanisms empower CSA-NET to adaptively focus on both high-level abstract features and fine-grained details that are critical for mammogram classification.

The post-attention mechanism processing features undergo aggregation via global average pooling (GAP), enabling the integration of spatial information across the entire image. Regularization techniques like dropout are applied to prevent overfitting, hence bolstering the model’s generalization capabilities.

The optimization of CSA-NET employs the NADAM optimizer with the focal loss function to mitigate class imbalances, steering the model learning towards minority classes. Training strategies such as early stopping and adaptive learning rate adjustments ensure efficient convergence and prevent overfitting.

The evaluation metrics encompass a comprehensive range including the accuracy, precision, recall, sensitivity, ROC curve, and F1-score. These metrics collectively reflect CSA-NET’s effectiveness in classifying both mammogram and ultrasound images, underscoring its potential to advance breast cancer diagnosis through early detection and precise characterization (Algorithm 1).
**Algorithm 1 **Algorithm of Proposed Model**Input:** *E*: Number of Epochs; α: Learning Rate; *b*: Batch Size; Breast cancer classification: $X_{train}$: Training dataset; $X_{test}$: Test dataset;**Output:** $A_{test}$: Classification evaluation scores on test dataset**Data Preprocessing:** $X_{train}\leftarrow Pre-process(X_{train}$), $X_{test}\leftarrow Pre-process(X_{test}$)**Training of Breast Cancer Classification:** $\delta \leftarrow DL-Model(X_{train},E)$, **while** δ has not converged **do**  **for** local epoche ϵ←1 to *E* **do**   **for** b=(x,y)∈ random batch from $X_{train}$ **do**    Apply Channel and Spatial Attention    Update the breast classification model weights and biases    $\delta_{i}\leftarrow \delta_{i}-\alpha (\Delta (L(\delta_{i}$;b)))   **end for**  **end for** **end while** Evaluation Scores of Breast Cancer Classification Model $A_{test}\leftarrow calculateScore\left(\delta ,X_{test}\right)$**return:** $A_{test}$

**Table 1:** Baseline model selection.

Network	Mean Av. Precision	Mean Av. Recall	Mean Av. Accuracy	Mean Av. ROC	Mean Av. Specificity	Mean Av. F1-Score
DenseNet-121 [54]	0.522	0.541	0.541	0.693	0.847	0.531
DenseNet-169 [54]	0.461	0.475	0.475	0.631	0.825	0.468
DenseNet-201 [54]	0.508	0.534	0.534	0.691	0.845	0.521
ResNet-101 [55]	0.404	0.435	0.435	0.500	0.812	0.419
ResNet152V2 [55]	0.401	0.506	0.506	0.468	0.835	0.447
InceptionResNetV2 [56]	0.530	0.533	0.533	0.700	0.844	0.531
EfficientNet-B0 [53]	0.744	0.748	0.748	0.905	0.916	0.746
EfficientNet-B1 [53]	0.734	0.736	0.736	0.909	0.912	0.735
EfficientNetV2-B0 [53]	0.610	0.620	0.620	0.832	0.873	0.615


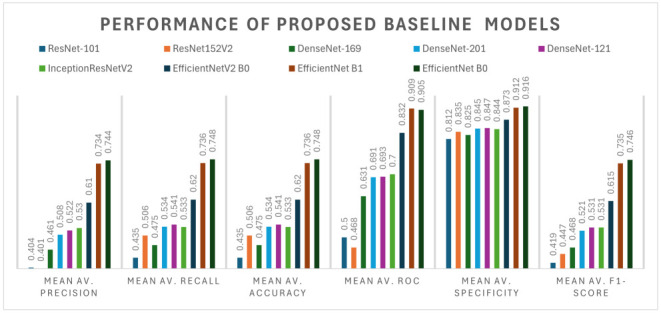


#### 3.1.1. Channel and Spatial Attention Mechanisms

**Figure 3:**
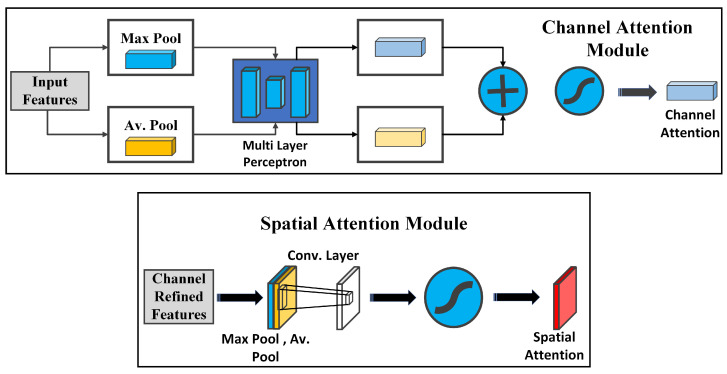
Channel and spatial attention mechanisms.

The CBAM block leverages both channel and spatial attention mechanisms; see Figure 3. Channel attention Equation (1) is executed by applying shared dense layers to the global average-pooled and global max-pooled representations of the input feature. The resulting attention weights are combined and applied to the input feature. Spatial attention—see Equation (2)—begins by concatenating the average- and max-pooled representations, followed by a convolutional layer that generates spatial attention weights. The input feature is then multiplied by these weights, allowing the model to focus on crucial spatial regions [57].

$$M_{ch}\left(F\right)=\sigma \left(MLP_{ch}\left(Av.Pool\left(F\right)\right)+MLP_{ch}\left(Max.Pool\left(F\right)\right)\right)$$

(1) $$=\sigma (W_{1_{ch}}\left(W_{0_{ch}}\left(F_{av}^{c}\right)\right)+W_{1_{ch}}\left(W_{0_{ch}}\left(F_{max}^{c}\right)\right))$$

where σ refers to the sigmoid function, $W_{0_{ch}}\in R^{C/r\times C}$ and $W_{1_{ch}}\in R^{C\times C/r}$. Here, $W_{0_{ch}}$ and $W_{1_{ch}}$ are the weights of the multi-layer perceptron (MLP) and are shared for both inputs. Additionally, the ReLU activation function is applied following $W_{0_{ch}}$.

$$M_{sp}\left(F\right)=\sigma \left(f^{3\times 3}\left(\left[Av.Pool\left(F\right);Max.Pool\left(F\right)\right]\right)\right)$$

(2) $$M_{sp}\left(F\right)=\sigma \left(f^{3\times 3}\left(\left[F_{av}^{s};F_{max}^{s}\right]\right)\right)$$

where σ is the sigmoid function, and a filter size of 3 × 3 is employed for the convolution operation $f^{3\times 3}$.

This comprehensive architecture is precisely designed to reinforce the model’s capacity to capture relevant features through the strategic incorporation of spatial and channel attention techniques. The overall objective is to elevate the performance in tasks such as breast cancer classification. See Figure 2.

#### 3.1.2. Loss Function

Focal loss serves as a powerful tool to address the class imbalance by assigning distinct weights to different classes, and its behavior is controlled by the parameters alpha and gamma. The focal loss function [31] is defined in Equation (3):

(3) $$Loss=-\frac{1}{B}\sum_{i=1}^{B}{\left(1-pl\left(s_{i}\right)\right)}^{\gamma }\log\left(pl\left(s_{i}\right)\right)$$

where *i* indicates the index of the *i*th sample within a batch of size *B*, and the final dense layer outputs the probability *p* for the sample $s_{i}$. The ground truth is denoted by *l*, representing the predicted value. Here, γ is a test sample’s weighting factor, setting its value to 2.

## 4. Results

In this section, the results that are generated by the proposed CSA-Net model are first discussed and then evaluated against the state-of-the-art models.

### 4.1. Materials

This section provides an overview of the datasets utilized, the experimental setup employed, the data augmentation techniques applied, and the evaluation measures used in our study. We will investigate each of these aspects to offer a comprehensive understanding of the methodology applied.

#### 4.1.1. Dataset

This section covers the details of the datasets used for this study. The datasets include INbreast [58], MIAS [59], CBIS-DDSM [60], and the combination of CBIS-DDSM, MIAS, and INbreast (CIM) [61]. The reason for using more than one dataset is to verify the effectiveness of the proposed methodology and compare its results on multiple datasets.

A description of each dataset is given in Table 2.

The datasets CBIS-DDSM, MIAS, and INbreast are composed of Dicom images. For the INbreast dataset, out of 410 case images, 106 mass cases were selected, preprocessed, and augmented. The images were preprocessed using CLAHE and augmented to a total of 7632 images of binary classes [61]. Each image belonged to the binary class of benign or malignant breast cancer. The numbers of images in the benign and malignant classes after augmentation were 2520 and 5112, respectively. The same procedure was applied for the selection of images in the CBIS-DDSM and MIAS datasets.

The Breast Ultrasound dataset [62] comprises three classes: normal, benign, and malignant. A total of 781 images are included, with 134 images for normal, 437 for benign, and 210 for malignant. The dataset is used for the cross-modality multi-class classification of the proposed method.

Summarizing this dataset overview, we provide an initial image distribution across classes in the multi-class datasets before any augmentation techniques are applied.

#### 4.1.2. Experimental Setup

This research explores the application of deep learning alongside enhanced data preprocessing techniques for breast cancer classification using benchmark datasets. The issue of class imbalance is addressed by using the upsampling approach. The study balanced minority classes by replicating images, ensuring equitable representation in the training set. The dataset is then divided into 60% for training, 20% for validation, and 20% for testing.

During the training phase, the focal loss [63] and the Nadam optimizer are utilized for 50 epochs. To counteract overfitting, early stopping is incorporated. Furthermore, the learning rate is dynamically adjusted based on the validation performance, beginning at 0.001 and decreasing by a factor of 0.25 after every five epochs. The backend framework utilized in this study is Keras 3.2.1 and TensorFlow 2.17.0, while an NVIDIA K80 GPU is employed for the training of different model configurations.

To optimize the training process, the focal loss function is adopted as in [31]. The focal loss proves advantageous in addressing class imbalances and classifying samples, making it particularly well suited for medical image classification tasks like breast cancer diagnosis.

In terms of optimization, the Nadam optimizer is selected, blending the merits of the Adam and Nesterov accelerated gradient techniques. Renowned for its efficiency in training deep neural networks, the Nadam optimizer facilitates enhanced generalization and accelerated convergence.

To augment the diversity of the training dataset and fortify the model’s resilience and generalization capabilities, various data augmentation strategies are introduced. These include random rotations, flips, zooms, and shifts, contributing to dataset enrichment and mitigating the risk of overfitting.

#### 4.1.3. Data Augmentation

The data augmentation process incorporates a suite of transformations aimed at enriching the training dataset. The specific augmentation parameters include a rotation range of 180 degrees, allowing for comprehensive adjustments in the image orientation. To accommodate slight variations in object positioning, a width shift range of 0.1 and a height shift range of 0.1 are introduced for modest translations in the vertical and horizontal positions, thus enhancing the model’s adaptability. The inclusion of a zoom range of 0.1 enables the diverse scaling of the images, leading to dataset variability. Horizontal and vertical flips are both set to True, introducing additional image variations by mirroring along the corresponding axes. The ’nearest’ fill mode ensures that any voids resulting from these transformations are filled with the closest pixel values available. Collectively, these augmentation techniques—rotation, shifting, zooming, and flipping—contribute to an augmented dataset with increased diversity, reinforcing the model’s capacity for generalization and its long-term reliability.

Addressing the issue of limited data, a data augmentation strategy is employed. This strategy includes random rotations ranging from 0 to 180 degrees, horizontal and vertical flips, and adjustments in width, height, zoom, and brightness. Subsequently, image resolution is standardized to 224 × 224 pixels.

#### 4.1.4. Evaluation Measures

Whenever a model undergoes training, its efficiency requires some means of measurement. Based on this, the model is evaluated as efficient or not. To evaluate the model’s efficiency, the trained model is tested for its precision, recall, accuracy, specificity, and area under the curve (AUC). These efficiency measurement mechanisms are further dependent on the prediction outcomes of the confusion matrices for breast cancer images: true positive (${\mathrm{TP}}_{B}$), true negative (${\mathrm{TN}}_{B}$), false positive (${\mathrm{FP}}_{B}$), and false negative (${\mathrm{FN}}_{B}$). The evaluation measures mentioned above provide results based on certain criteria, as explained below.

Accuracy: The accuracy is the number of true predicted classification instances out of the total occurred instances [64]. It is calculated as seen in Equation (4):

(4) $$Accuracy=\frac{{\mathrm{TP}}_{B}+{\mathrm{TN}}_{B}}{{\mathrm{TP}}_{B}+{\mathrm{TN}}_{B}+{\mathrm{FP}}_{B}+{\mathrm{FN}}_{B}}$$

Precision: Precision is the ability of the model to avoid false negatives. It is the ratio of true positive (${\mathrm{TP}}_{B}$) or correctly positively classified predictions to all positive predictions [64]. See Equation (5). Its calculation plays a vital role, especially in medical diagnostics, due to identifying false positive (${\mathrm{FP}}_{B}$) cases. It is worth mentioning that the effect of minimizing the FP is valuable in many aspects of the process of diagnosis, from costs to diagnostic procedural complexity.

(5) $$Precision=\frac{{\mathrm{TP}}_{B}}{{\mathrm{TP}}_{B}+{\mathrm{FP}}_{B}}$$

Sensitivity or Recall: Sensitivity is a measure of the true positive predictions that are positives; see Equation (6). The success of a model in identifying a positive case is reflected by the sensitivity. This measure is quite helpful in minimizing false negative cases [64].

(6) $$Sensitivity=\frac{{\mathrm{TP}}_{B}}{{\mathrm{TP}}_{B}+{\mathrm{FN}}_{B}}$$

Specificity: Specificity is defined as the fraction of true negatives accurately predicted by the model. It quantifies the model’s ability to correctly identify instances that do not belong to the positive class, offering a measure of its accuracy in recognizing true negatives within the dataset [64]. See Equation (7):

(7) $$Specificity=\frac{{\mathrm{TN}}_{B}}{{\mathrm{TN}}_{B}+{\mathrm{FP}}_{B}}$$

F1-score: The F1-score, also known as the F-measure, serves as a pivotal metric in evaluating the effectiveness of classification models, particularly in scenarios involving binary classification tasks. The F1-score, which combines the precision and recall, offers a balanced assessment of the model’s performance, ensuring a fair evaluation of both its accuracy and reliability.

(8) $$F1-Score=2\times \frac{\mathrm{Precision}\times \mathrm{Recall}}{\mathrm{Precision}+\mathrm{Recall}}$$

Area Under the Curve (AUC): The receiver operating characteristic (ROC) curve’s assessment outcome, commonly denoted by the area under the curve (AUC), provides valuable insights into the model’s performance. The AUC serves as a key performance metric for classification models, providing insights into their effectiveness across different threshold values. This metric is instrumental in gauging the model’s discriminatory power and overall performance in classifying instances within the dataset [64].

### 4.2. Ablation Study

The ablation study integrates three diverse datasets—CBIS-DDSM, INbreast, and MIAS—for distinct tasks within the realm of medical image classification. EfficientNet-B0 [53] is the base model used to check the performance of the binary and multi-class classification on mammogram images. The model has undergone a three-fold training procedure, and the performance evaluation of the base model is described in Table 3.


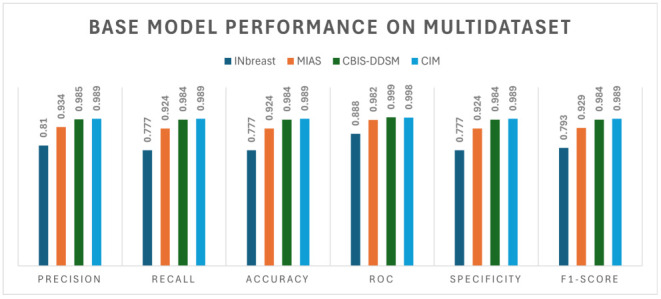


#### 4.2.1. Binary Classification

An ablation study is conducted to enhance the generalization of the proposed model, thus resulting in the reinforcement of the robustness and diversity of the training dataset. This study involves two classifications, utilizing the datasets CBIS-DDSM, INbreast, and MIAS for two classes, benign and malignant. Due to the uneven distribution of the classes in the experiments, we employ data upsampling techniques. Additionally, data augmentation methods are applied, including random rotation and vertical and horizontal flipping. For optimization during backpropagation, the Nadam method is considered. In our investigation of the base models, we found that a pre-trained EfficientNet-B0 [53] network achieved the highest accuracy score among all examined models; refer to Table 1. Consequently, we use EfficientNet-B0 as the base model to evaluate the performance of binary and multi-class classification on mammogram images.

#### 4.2.2. Cross-Modality Multi-Class Classification Results Using Ultrasound Images

This study incorporates ultrasound images from the Breast Ultrasound dataset [62] for a cross-modality analysis. This analysis necessitates evaluating the model’s generalization capabilities across different imaging modalities, specifically ultrasound and mammography. The model is trained on mammography images from the CBIS-DDSM, INbreast, MIAS, and CIM datasets and subjected to evaluation using ultrasound images to assess its robustness and performance in a cross-modality context. The outcomes obtained from this evaluation offer valuable insights into the model’s capacity for cross-modality generalization, providing crucial information about its adaptability and effectiveness when applied to ultrasound images, in addition to its original training modality of mammography.

This comprehensive investigation strategically leverages the strengths inherent in each imaging modality and dataset to address specific challenges in medical image classification. Through the incorporation of diverse datasets, the model gains exposure to intricate patterns associated with various imaging modalities and clinical scenarios. This holistic approach culminates in the development of a more adaptable and reliable classification model tailored to the assessment of breast health. The amalgamation of information from different sources enhances the model’s capacity to discern refined features and variations, thus contributing to a robust and versatile solution for medical image analysis in the context of breast health assessment.

## 5. Discussion

### 5.1. Comparison of Results before and after Using Attention Mechanism

Analyzing the effect on the outcomes before and after the integration of the attention mechanisms yields significant insights into the influence of the mechanisms on the model’s overall performance. The attention mechanism is intricately crafted to augment the model’s focus on particular regions or features within the input data, with the potential to enhance its efficiency in capturing pertinent information. This comparative evaluation allows for a detailed understanding of how the attention mechanisms contribute to the model’s ability to discern and prioritize crucial aspects within the dataset, ultimately influencing its overall performance.

Table 4 presents a comparative analysis of the performance metrics across different datasets with and without the implementation of the channel and spatial attention (CSA) mechanism. Specifically, the datasets examined include Inbreast, MIAS, CBIS-DDSM, and CIM (comprising a combination of the MIAS, CBIS-DDSM, and INbreast datasets). Evaluation metrics such as the precision, recall, accuracy, ROC, specificity, and F1-score are considered for each dataset.

The results’ analysis indicates a consistent enhancement in performance across all datasets when CSA is applied. Notably, on the Inbreast dataset, CSA leads to improvements in accuracy, sensitivity, and precision. Similarly, significant enhancements are observed in the MIAS and CBIS-DDSM datasets, particularly in parameters like the ROC score, sensitivity, and precision.

Moreover, when evaluating the combined CIM dataset, the benefits of CSA become more pronounced, with notable improvements in the ROC score, accuracy, sensitivity, and precision. These findings underscore the effectiveness of the CSA mechanism in augmenting the model’s capability to extract crucial spatial and channel-wise features, consequently boosting the classification performance across diverse datasets. Overall, the inclusion of channel and spatial attention contributes to enhanced classification accuracy and model robustness across multiple datasets.

**Table 4:** Performance evaluation of proposed model with and without attention mechanism on binary datasets.

Dataset	Mean Av. Precision	Mean Av. Recall	Mean Av. Accuracy	Mean Av. ROC	Mean Av. Specificity	Mean Av. F1-Score
Without Attention						
Inbreast	0.810	0.777	0.777	0.888	0.777	0.793
MIAS	0.867	0.867	0.867	0.932	0.867	0.867
CBIS-DDSM	0.985	0.984	0.984	0.999	0.984	0.984
CIM	0.989	0.989	0.989	0.998	0.989	0.989
With Attention ^1^						
Inbreast	0.989	0.988	0.988	0.999	0.988	0.989
MIAS	0.956	0.954	0.954	0.994	0.954	0.955
CBIS-DDSM	0.999	0.999	0.999	0.999	0.999	0.999
CIM	0.999	0.999	0.999	0.999	0.999	0.999

^1^ CSA: Channel and Spatial Attention.


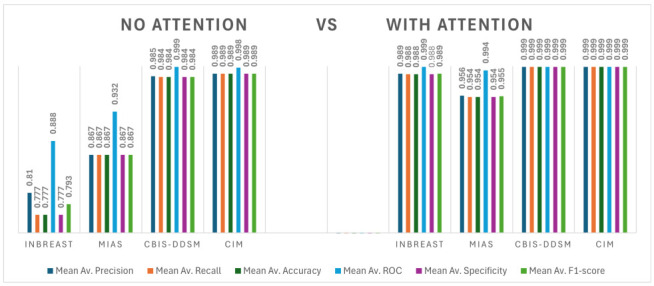


An important outcome after implementing CSA is the notable improvement in the accuracy, sensitivity, and F1-score across all datasets. By selectively attending to meaningful channels and spatial locations, the model becomes more proficient in detecting significant patterns and refinements in the data, resulting in more accurate classifications. Specifically, in datasets like MIAS and CBIS-DDSM, where the precision and F1-score values approach perfection after applying CSA, this enhancement is particularly striking.

Furthermore, utilizing CSA enhances the model’s overall performance and discriminatory ability by improving the accuracy and area under the ROC curve (AUC). The achievement of near-perfect accuracy when employing CSA on the CBIS-DDSM dataset suggests that the attention mechanisms effectively capture the complex patterns and variations present in the data.

Moreover, the incorporation of CSA improves the model’s generalization and resilience across diverse datasets. By focusing on both channel-wise and spatial-wise features, the model becomes more robust to the variations and complexities inherent in the different datasets, resulting in consistent performance improvements across datasets like INbreast, MIAS, and CBIS-DDSM.

**Figure 4:**
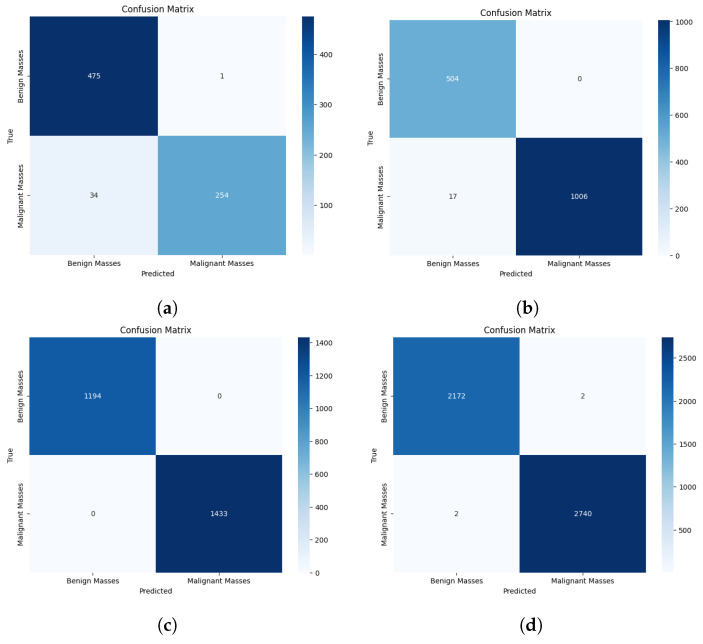
Confusion matrix of proposed model on different datasets. (**a**) Confusion matrix of MIAS dataset. (**b**) Confusion matrix of INbreast dataset. (**c**) Confusion matrix of CBIS-DDSM dataset. (**d**) Confusion matrix of CIM dataset.

**Figure 5:**
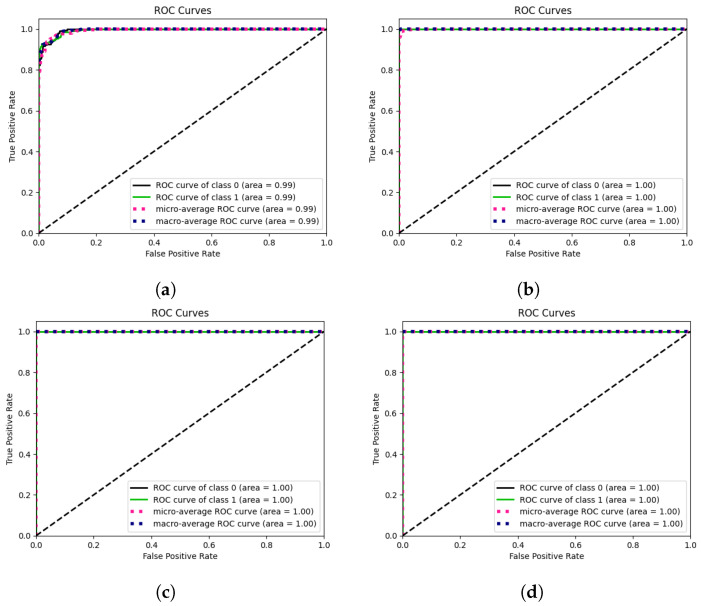
ROC of proposed model on different datasets. (**a**) ROC on MIAS dataset. (**b**) ROC on INbreast dataset. (**c**) ROC on CBIS-DDSM dataset. (**d**) ROC on CIM dataset.

In this study, we conduct a thorough comparison of the proposed CSA-Net approach with several existing breast cancer diagnosis methods across three datasets: CBIS-DDSM, INbreast, and MIAS (Table 5 and Figure 4 and Figure 5). To ensure a fair and accurate comparison, identical experimental setups on all datasets are utilized. Additionally, comprehensive performance measures are provided, including the F1-score, ROC, accuracy, precision, recall, and specificity.


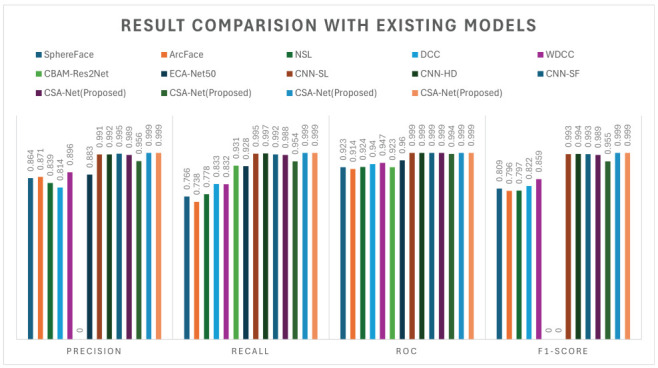


Through multiple experiments conducted on three publicly accessible datasets, our proposed approach consistently shows improvements in its classification performance. Notably, the integration of the spatial and channel-wise attention mechanisms results in state-of-the-art outcomes, further validating the effectiveness of the proposed approach.

### 5.2. Cross-Modality Multi-Class Classification Results Using Breast Ultrasound Dataset

The results provided in Table 6 showcase the model’s performance on the Breast Ultrasound dataset, encompassing three distinct classes: normal, benign, and malignant. The model demonstrates commendable results, achieving 92.9% precision and 92.3% recall, indicating its capacity to identify accurately positive instances while minimizing false positives. With overall accuracy of 92.3%, the model showcases reliable classification across the dataset. The high ROC score of 96.0% underscores the model’s proficiency in distinguishing between classes. Additionally, the specificity score of 96.1% reflects the model’s effectiveness in correctly identifying true negative cases. These results, combined with the F1-score of 92.1%, affirm the model’s robust performance in accurately classifying breast lesions in ultrasound images across diverse categories.

**Figure 6:**
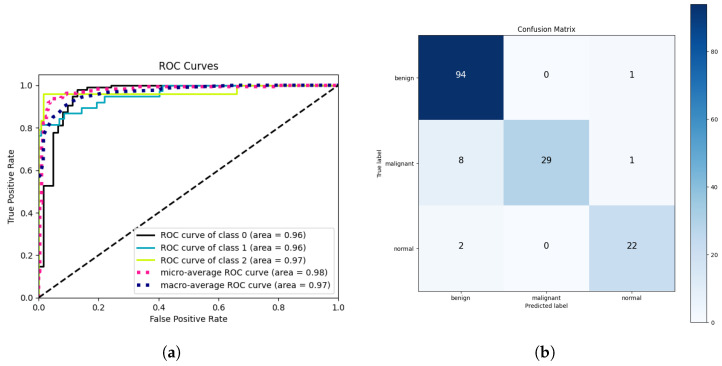
ROC graph and confusion matrix for multi-class cross-modality. (**a**) ROC on Breast Ultrasound dataset. (**b**) Confusion matrix of Breast Ultrasound dataset.

CSA-Net achieves high classification accuracy, as evident in Figure 6, indicating that it effectively pinpoints lesions and assigns them to the appropriate categories. However, there are instances where the expected samples do not yield accurate predictions. In cases where only one lesion is present among the incorrectly predicted samples, our approach typically prioritizes the lesion area for classification.

## 6. Conclusions

This study illustrates the efficiency of employing a deep learning model with channel and spatial attention mechanisms for breast cancer classification. By achieving promising outcomes on both mammogram and ultrasound datasets, the model exhibits potential for practical implementation in clinical settings. In binary classification tasks, the model shows notable improvements, especially upon the integration of channel and spatial attention mechanisms, reaching 99.9% accuracy on a combined dataset of CBIS-DDSM, INbreast, MIAS, and CIM. This enhancement emphasizes the model’s capacity to discern critical features within the data, thereby enhancing its classification performance. The model’s versatility across different imaging modalities is evident in its successful evaluation with breast cancer ultrasound images. This capability highlights the model’s adaptability and reliability in varying clinical scenarios.

Through a cross-modality multi-class analysis involving breast cancer ultrasound images, the model demonstrates its proficiency in generalizing across diverse imaging modalities, showing accuracy of 92.3% on three classes. Its high precision, recall, accuracy, ROC score, and specificity collectively affirm its robustness in accurately classifying breast lesions, further solidifying its potential in real-world clinical applications.

## Figures and Tables

**Table 2:** Details of binary class datasets.

Dataset	Classes	Class Names	Total Images	Benign	Malignant
INbreast	2	BM/MM	7632	2520	5112
MIAS	2	BM/MM	3816	2376	1440
CIM ^1^	2	BM/MM	24,576	10,866	13,710
CBIS-DDSM	2	BM/MM ^2^	13,128	5970	7158

^1^ CIM: CBIS-DDSM + INbreast + MIAS datasets. ^2^ BM: Benign Mass, MM: Malignant Mass.

**Table 3:** Performance evaluation of base model.

Dataset	Classes	Class Names	Precision	Recall	Accuracy	ROC	Specificity
INbreast	2	BM/MM ^2^	0.810	0.777	0.777	0.888	0.777
MIAS	2	BM/MM	0.934	0.924	0.924	0.982	0.924
CBIS-DDSM	2	BM/MM	0.985	0.984	0.984	0.999	0.984
CIM ^1^	2	BM/MM	0.989	0.989	0.989	0.998	0.989

^1^ CIM: CBIS-DDSM + INbreast + MIAS datasets. ^2^ BM: Benign Mass, MM: Malignant Mass.

**Table 5:** Result comparison of binary classification with existing models.

Citation	Dataset	Precision	Recall	Accuracy	ROC	Specificity	F1-Score
SphereFace [20]	INbreast	0.864	0.766	0.912	0.923	-	0.809
ArcFace [21]	INbreast	0.871	0.738	0.912	0.914	-	0.796
NSL [22]	INbreast	0.839	0.778	0.905	0.924	-	0.797
DCC [23]	INbreast	0.814	0.833	0.912	0.940	-	0.822
WDCC [23]	INbreast	0.896	0.832	0.934	0.947	-	0.859
CBAM-Res2Net [34]	INbreast/DDSM	-	0.931	0.933	0.923	-	-
ECA-Net50 [35]	INbreast	0.883	0.928	0.929	0.960	-	-
CNN-SL [36]	DMR-IR	0.991	0.995	0.993	0.999	0.995	0.993
CNN-HD [36]	DMR-IR	0.992	0.997	0.994	0.999	0.997	0.994
CNN-SF [36]	DMR-IR	0.995	0.992	0.993	0.999	0.992	0.993
CSA-Net	INbreast	0.989	0.988	0.988	0.999	0.988	0.989
CSA-Net	MIAS	0.956	0.954	0.954	0.994	0.954	0.955
CSA-Net	CBIS-DDSM	0.999	0.999	0.999	0.999	0.999	0.999
CSA-Net	CIM	0.999	0.999	0.999	0.999	0.999	0.999

**Table 6:** Cross-modality results on Breast Ultrasound dataset.

Dataset	Classes	Precision	Recall	Accuracy	ROC	Specificity	F1-Score
Breast Ultrasound	3	0.929	0.923	0.923	0.960	0.961	0.921

## Data Availability

The datasets include INbreast [58], MIAS [59], CBIS-DDSM [60], and the combination of CBIS-DDSM, MIAS, and INbreast (CIM) [61].
